# “We are doing the best we can to bridge the gap” - service provider perspectives of educational services for autism spectrum disorder in South Africa

**DOI:** 10.3389/fpsyt.2022.907093

**Published:** 2022-07-19

**Authors:** Sarosha Pillay, Madeleine Duncan, Petrus J. de Vries

**Affiliations:** ^1^Department of Health and Rehabilitation Sciences, University of Cape Town, Cape Town, South Africa; ^2^Centre for Autism Research in Africa, Division of Child & Adolescent Psychiatry, University of Cape Town, Cape Town, South Africa

**Keywords:** autism spectrum disorder, service provider perspectives, education systems, low-and- middle-income countries, service delivery

## Abstract

**Background:**

The South African education system is increasingly unable to meet the growing needs of children with autism spectrum disorder (ASD). Recent studies in the Western Cape, one of the better resourced provinces in South Africa, showed that the pathway to care for children with ASD was an inconsistent and lengthy process, and that many children with ASD waited for extended periods to get access to an appropriate school placement. It is therefore clear that scalable and sustainable solutions are required to improve access to appropriate education for children with ASD.

**Methods:**

Here we performed a qualitative study using thematic analysis of ten multi-sectorial ASD service provider interviews in the Western Cape Province to examine provider perspectives and proposed solutions to meet the educational needs of children with ASD.

**Results:**

Provider perspectives were grouped in three categories: “bridging the gap across the spectrum and lifespan”, “gaps to bridge”, and “building bridges”. The first category captured provider perspectives of the service-related needs inherent to a diagnosis of ASD. The second category summarized service provider views of the challenges associated with providing services to children with ASD and the third category captured provider perspectives on potential actions to improve ASD education services delivery in the province. The overarching theme that emerged was “We're doing the best we can to bridge the gap”.

**Conclusion:**

Participants provided ten key recommendations for service strengthening that may lead to contextually relevant innovations to meet the educational needs of children with ASD in the province. Findings from this study has direct relevance to other South African provinces and may have relevance to improve pathways and reduce service delivery gaps also in other low-and-middle-income countries.

## Introduction

Autism Spectrum Disorder (ASD) is a complex developmental disorder that affects 1–2% of the world's population at varying degrees and is characterized by a range of impairments in the areas of social communication, learning and behavior (1). The quality of life of many individuals with ASD and their families are significantly impacted by these impairments (2–4) and individuals with ASD may require services across the lifespan to minimize and manage some of the core features of ASD and co-occurring conditions (5, 6).

High-income countries (HIC) are typically better resourced to meet the needs of individuals with ASD and their families. However, even in some HIC there have been reports on challenges in service delivery, most notably in the areas of access to early diagnostic evaluations (7), policy implications for early intervention and support for school-aged children with ASD (8), and inclusion and employment of individuals with ASD (9).

Olusanya et al. (10, 11) reported that 95% of all children with developmental disabilities including ASD live in low-or middle-income countries (LMIC) (10, 11), yet there is little information on how these children are identified, evaluated, treated and educated (12–15). In a scoping review of all autism research in sub-Saharan Africa, Franz and colleagues identified that less than 1% of the world's autism research had taken place in Africa, and that no studies had examined health or education systems for children with autism (15).

South Africa has a population of 58.8 million people of diverse cultural and socio-economic backgrounds (16). It is an upper-middle income country with the highest Gini co-efficient indicating vast socio-economic disparities between rich and poor (17). High Gini co-efficient is characteristic of many LMICs, including India and most Latin-American countries (18). South Africa therefore has a socio-economic profile that is very representative of the needs of the majority of the world's population.

Vast disparities across social class and racial lines exist in access to public health and education services due, amongst others, to the socio-political legacy of apartheid (19–21). In South Africa, children with disabilities including those diagnosed with ASD are most at risk of not having their health, social and educational needs met due to reliance on state-funded services used by the majority of the population (22). The South African special education sector is a product of the apartheid era where children were historically classified according to race and disability (22) and children with specific disabilities could only be enrolled at the few available schools allocated to the disability. Although there have been efforts to correct these legacies of apartheid, in 2016 there were 119 403 children with disabilities attending 455 schools across the country (23) and an estimated 597,953 children with disabilities reported to be out of schools (24). The limited state-funded services for children with special education needs in South Africa therefore tend to be oversubscribed resulting in long periods of waiting for access.

In a study of the educational system for ASD in the Western Cape Province of South Africa, Pillay and colleagues (25) set out to identify all school-aged children with a diagnosis of ASD in the Western Cape Education Department database. Out of more than 1 million children, only 940 children with a diagnosis of ASD were identified, representing a rate of 0.08%. Based on a conservative ASD prevalence at 1%, the finding suggested a more than 10-fold under-identification of ASD in schools in the Western Cape province (25). Apart from the low numbers, the authors also identified very low rates of co-occurring diagnoses in the sample, complex and confusing pathways to diagnosis and treatment, and, surprisingly, found that 89% of children with an ASD diagnosis were in special educational placements (25). The authors next proceeded to search for those children waiting for a school placement in the province, and identified 744 children, with 266 (36%) of them being of legal school-going age, but not in education (26). Fifty two percent (52%) of children had been waiting for schools for more than a year (26). To compound the emerging picture of ASD in the province, the authors found a 76% increase in children with ASD in school between 2012 and 2016 (25), and a 276% increase in children on the “waiting list” for the same period (26). The findings from these earlier studies made it clear that, even in one of the better-resourced provinces of South Africa, the educational system was not able to meet the needs of children with ASD and their families.

In health systems research, Gilson and colleagues (27) pointed to the importance of understanding any given system in a “whole-system” way, including the “hardware” elements (e.g., human resources, infrastructure, financing), the ‘*s*oftware” elements (e.g., ideas and interests, relationships and power, values and norms), and the interaction between these hardware and software elements. Applying the whole-system concept to the educational system in the Western Cape province of South Africa, and in the context of previous work by Pillay and colleagues on hardware elements, we recognized the importance of exploring also the software elements in the education system in order to generate strategies that may support strengthening of the system. Pillay and colleagues (25, 26) examination of the hardware elements of the education system reported the rates, demographic, disability and educational profile of children with ASD both in schools and those waiting for schools in the province. These data provided a starting point for understanding the landscape of ASD education in the Western Cape and the authors proposed that engagement with stakeholders would be necessary next steps for developing a more comprehensive picture of the ASD situation in the province.

In an effort to complement the “hardware” findings of Pillay and colleagues, we therefore set out to examine the ‘*s*ocial” elements of the system by seeking the perspectives of service providers, a key stakeholder group, in the Western Cape province. Our overarching aim was to describe their views of existing services for children with ASD in the Western Cape, and their recommendations for improvements to existing service systems.

## Methods

### Design

A qualitative pragmatic research methodology (28) was used to collect descriptive data arising from the realities faced by ASD service providers.

### Participants and procedures

Purposive and snowball sampling was used to identify and recruit a broad range of service providers from the government, private and voluntary sectors. To be eligible, potential participants had to have first-hand experience of delivering ASD educational and other services, and knowledge of the waiting list for these services in the Western Cape province. Participants were invited by email or telephonically to participate. Written informed consent was obtained from all participants.

### Data collection

Individual semi-structured interviews of approximately 45–60 min were conducted by the first author. Interviews were conducted in English and were digitally recorded. The interview guide included broad, open-ended questions around service provider perspectives of existing services in the province and their proposed solutions to improve ASD service delivery. Clarification probes were used to ensure the following research questions were being answered: “what do service providers think about the current unmet education needs of children with ASD in the Western Cape province and what recommendations do they have for addressing these needs?” The interview was terminated when the interviewer and interviewee felt that data saturation was reached.

### Data analysis

Audio-recorded interviews yielded 7 h and 30 min of data that were transcribed verbatim by the first author into textual form for thematic analysis (29). NVivo version 12 was used for data storage, management and first level inductive coding to identify units of meaning expressed by the service providers. Second level coding of inductively identified codes was done manually and involved grouping codes into sub-categories and categories of meaning from which an overarching theme emerged. Thematic synthesis described by Thomas and Harden (30) was used where the primary researcher identified the sub-categories, categories, and theme and consensus was reached through discussions with the second author, an experienced qualitative researcher. All proposed subcategories, categories and themes were then presented to the third author for discussion until consensus was reached.

### Scientific rigor

The creditability and transferability of the data (31) were enhanced by the primary researcher's prolonged engagement and participant observation in the field that enabled the pertinent use of probes during the data gathering interviews. Participants verified the accuracy of the information reflected in the findings through a rigorous process of member checking (32). Rigor was also enhanced through data triangulation from different sources namely interviews, researcher field notes and document reviews.

### Ethical approval

Ethical approval was granted by the University of Cape Town Human Research Ethics Committee (HREC reference: 072/2016) as well as the Western Cape Department of Education (reference: 20150727-1712).

### Community participation

No individuals with ASD or their family members were directly involved in this study. Ten service providers who work with individuals with ASD and their families were involved.

## Results

### Demographic characteristics of participants

The characteristics of included participants are shown in Table 1. A total of ten participants across different professional groups, sectors, departments and base for work (urban/rural) were included.

**Table 1:** Participant characteristics.

**Participant** **number**	**Service provider**	**Sector**	**Urban/Rural**
P1	Medical doctor	Health	Urban
P2	Special school principal	Education	Urban
P3	Special school deputy principal	Education	Urban
P4	Occupational therapist	Education	Rural
P5	Educational psychologist	Education	Urban/Rural
P6	Educator	NGO	Urban/Rural
P7	Parent advocate	NGO	Urban
P8	ASD educational consultant	Private	Urban/Rural
P9	Medical doctor	Health	Urban
P10	Psychologist	Education	Urban/Rural

*NGO, non-government organization*.

### Thematic analysis

Table 2 provides a summary of the main theme, categories and sub-categories identified in analysis. The overarching theme that emerged in answering the research questions was “*we are doing the best we can to bridge the gap”*. The theme reflected service providers' awareness of the discrepancies that exist between the scope and nature of the ASD educational service demands and their capacity (either individually or collectively) to meet these demands. Providers expressed perspectives about the scope and nature of ASD service needs in three discreet capacity-related categories: (1) *bridging the gap across the spectrum and lifespan*, (2) *gaps to bridge*, and (3) *building bridges*. The first category captured provider perspectives of service-related factors inherent in the ASD diagnosis and intervention. That is, providers acknowledged the wide-ranging, lifelong and changing needs of children with ASD and their families. The second category summarized service provider views of the range of structural constraints that limited their capacity to meet service needs. The third category captured provider perspectives on potential actions to “build bridges” that might reduce the demand-capacity divide.

**Table 2:** Summary of main theme, categories and sub-categories.

**Theme**	**Categories**	**Sub-categories**	**Description of codes that constituted the sub-category**
We're doing the best we can to bridge the gap	Bridging the gap across the spectrum and lifespan	Lifespan factors	The lifespan needs of persons with ASD
		Disability factors	How the diagnostic label of ASD can be a barrier to services
		Curriculum factors	The fit between the learning potential and educational needs of each child with ASD and what they are being taught
		Policy factors	Participant views on the regulatory, policy and political factors influencing ASD services in the province
	The gaps to bridge	Resource constraints	The challenges in human and infrastructural resources
		Contextual constraints	Issues of litigation arising from unmet service needs, equity of access and the burden of public health care
		Competence constraints	The perceived competencies and skill-set of decision-makers, policy-makers and planners
	Building bridges	Leadership	The importance of leadership to address the challenges in ASD education
		Capacity building	Recommended actions that would strengthen the range and quality of resources
		Co-ordination	System efficiency through managed interagency collaboration
		Innovation	Novel actions for improved service delivery


The theme, categories and sub-categories as presented in Table 2 will next be discussed with representative quotes.

### Category 1: Bridging the gap across the spectrum and lifespan

Service providers spoke about the need for ASD services to be available to individuals with all levels of severity of ASD throughout the different stages of their life. Four sub-categories emerged: lifespan factors, disability factors, curriculum factors, and policy factors.

#### Lifespan factors

Given the lifespan implications of the diagnosis, service providers felt that the right type of service was essential to support the individual with ASD across the different stages of life and that these services were generally lacking:

“*That's what we need, cradle to grave provision…And I do think it would be great if the Western Cape could do that…if it was possible” (P8)*

#### Disability factors

This sub-category contained perspectives about the diagnosis of autism acting as an inherent barrier to service access and inclusion:

“*If I didn't know you were autistic, I might still provide services for you, but now, oh but you come with that label, oh you're ASD, sorry then you fall into that stream…You see, and for me, that is then a disservice to the child” (P9)*

“*The question was asked to me by somebody yesterday, about, why is autism so fearful? Everybody cringes when they hear you have to take learners with autism, why can't they just go to other schools?” (P2)*

Service providers stressed the right to participation in education for all children with ASD regardless of the severity of disability. Some expressed concern that ASD children presenting with high levels of needs were denied access to educational services because of the amount of individualized functional support that they require. These children are referred to “special care centers” (centers for children with severe-to-profound intellectual disability and associated ASD) where the education-related intervention that they receive is not always optimal:

“*…but the special care centers can't necessarily cater for our children with autism, who are very busy, and they don't present the same as the other children at the special care centers. The disability is just so different. So what happens to those children who can't be supported in an autism school, and needs even higher level of support?” (P5)*

#### Curriculum factors

The quality of education for children with ASD was a big concern with some service providers feeling that the curriculum did not prepare young adults with ASD for vocation after school:

“*… where we are focusing on skills, we're focusing on really mundane skills… And I think situations where our teenagers and our young adults being taught, for example, to put windscreen wipers into boxes... that's not a career” (P7)*

The need for differentiation of the mainstream curriculum to accommodate and optimally support the learning potential of all children with ASD according to their developmental needs was stressed:

“*I think that there might be a lot of children who are in autism schools, the traditional autism schools, who could potentially find a home in mainstream schools, if there was the willingness*

*to say “you know what? This child is not going to be attending English, it's going to go to Maths, and then he's going to sit in his or her own little space for the next hour”. I don't know, I mean obviously there are massive kind of logistical requirements, and you've got a schooling system already under strain…” (P10)*

#### Policy and political factors

This sub-category included perspectives on the moral and legislative right to education for all children irrespective of ability:

“*sorry, I take the extreme view, every child has a right to education in the constitution, and they should all be in school, and I know they're not…Everybody else can fight for their piece of cake, but we need to say, if we're not looking after the most needy citizens, then what are we as a society?” (P8)*

Concerns were raised that ignorance about the diagnosis and its service requirements rendered policy-makers ineffective. Service providers felt that government stakeholders and policy-makers did not have a good understanding of the ASD situation in the province and that people who did know (such as service providers, individuals with ASD and their families) were not included in policy decision-making processes, leading to “knee-jerk” short-term rather than strategic long-term actions:

“I think they just don't know. Honestly, my opinion is that the policy makers and even some of the people who are writing the adapted curriculums have never set foot in a classroom. Some of the people who are even writing the SIAS^*^ *document, for example, which I think is a brilliant document, have never stepped into a classroom. And you can't... you can't do this... you can't make decisions for the people on the ground if you've never been on the ground” (P7)*

^*^Policy on Screening, Identification, Assessment and Support (SIAS) provides a framework for standardized procedures to facilitate the inclusion of children who require additional support in schools.

“…*they* [policy makers] are *reactionary. I understand why, I understand the pressures that they experience and they have to endure, but they have knee-jerk reactions all the time” (P3)*

Perspectives on policy compliance and the political agenda behind policy development and implementation were raised. Service providers felt that the lack of transparency around the ASD waiting list had unfortunate consequences for the children with ASD:

“*I call it a political game as well…we can't get away from it…I'm not saying the one is better than the other* [political party], *but it's politics. And unfortunately, the children suffer. And once again it comes back to my point of, I question, is it in the best interest of the children?” (P3)*

“*I suspect that it had become something that would potentially be politically very, very uncomfortable, were it to arrive in the public domain; that in reality, that we were able somehow to generate a list of children who weren't in schools for autism, versus available proper spaces, it would look like a disaster.” (P10)*

### Category 2: Gaps to bridge

The second category described resource, contextual and competence constraints that were creating service gaps that needed to be bridged in order for ASD services in the Western Cape to meet current and future needs.

#### Resource constraints

Resource constraints referred to challenges in human and infrastructural resources, and the impact thereof on a) service provider wellbeing, and b) the ability of the education system to provide critical early intervention services.

With the growing demand for ASD educational services, service providers felt the current infrastructure was not keeping up with the need for customized physical space:

“*I don't see any significant moves, plans, to provide that infrastructure. And when I talk infrastructure, I mean the hard buildings, people, you know, not just a programme, but the physical facilities to provide in this growing need, that is just getting more and more. You know, we're already battling with a backlog, we're sitting with this backlog…But it's not just trying to catch up, we have to provide for the ever growing number” (P9)*

Human resources including people with the necessary skills and willingness to work with children with ASD was seen as a major gap that needs to be bridged:

“*I think what we're lacking is people. People qualified to work in it, and not just people qualified, people passionate about working in it. And I think this isn't a field to go into for a nine to five job. You go into this field, it's hard work, the kids do have their challenges, and as gorgeous as they are, they aren't without tough days, and what we're lacking is people who want to work with that. So, for me, that's the biggest resource we're missing, is people. People motivated to work in the field” (P7)*

In the absence of appropriate and adequate resources, it was felt that the increasing pressures put on existing ASD schools by the education department to place more children in a classroom to alleviate the waiting list would have negative consequences on the quality of education and on the mental health of the staff:

“*I'm concerned about the quality of education that we're going to deliver from here on in. I'm very concerned about that because our staff members will be burnt out.” (P3)*

Participants were of the opinion that resource constraints also contributed to the constant delays in establishing essential early intervention programmes. With the pressures that the education department faced in providing education for school-aged children with ASD, early intervention programmes were being neglected:

“*I think [it] is absolutely heart-breaking, never mind heart-breaking, it's also a human right being denied, in my opinion, that's early intervention… it's not available, because they need to deal with the waiting list.” (P8*)

“*I think the communication support for children, knowing that there's some critical windows, in young lives. And I'm talking about the two to four / five year age group, that missing period of communication support.” (P10)*

#### Contextual constraints

The context within which the growing ASD waiting list exists was highlighted. Service providers acknowledged that education for children with ASD was only one of the public health challenges that the education department was faced with:

“*But I think people are working hard, and I think one shouldn't underestimate the amount of work that the Education Department must be looking at, and even Early Childhood Development. Because remember, we're just looking at autism. We have huge numbers… I have children with cerebral palsy and other special needs, who are also struggling to get services” (P1)*

Some service providers indicated that the waiting list was a useful tool for monitoring the need for services “*it is a fair system*” (P3) while others questioned its purpose:

“*I think that initial idea with the waiting list, was... it was meant well, but it turned out to be a disaster. I think there exists, quite a huge misunderstanding, a lack of knowledge, regarding the… what is the waiting list? Waiting for what? Waiting for placement? Waiting for assessment? Waiting for a chair? What are we waiting for? And to me, it is sad... as much as I understand the need, that, we have to put people on a list to get the services to them, I disagree with them having to be on a list, especially the numbers that we're talking about…” (P10)*

Concerns about children on the waiting list not receiving any intervention while they wait for a placement at a school was raised:

“*And that confusion, I think, contributes to part of the problem. Some think, no they are being serviced somewhere, so they're okay. And then, yet there are others that are also on the waiting list, but they're sitting in the home, so they're out of school, and nothing happens with them, for years on end” (P9)*

Service providers felt that children from lower-income homes with parents who did not complain or who were foreign nationals were more at a disadvantage:

“I think there's been a huge move of people into the Western Cape. And wherever you see immigrant groups, large numbers of children from the DRC^*^, there's been children from Somalia, and they are all trying to access services. These children are born in this country, so it's not a case that they've come from other places to access services, they were born here, but they are needing services and education. So I think we are having to extend the number… or increase the number of… or capacity of autism units” (P1)

^*^ *DRC – Democratic Republic of Congo*

#### Competency constraints

Participants attributed the current situation in service delivery to lack of capacity rooted in ignorance, denial and poor planning. The competence of decision makers in assessing the urgency of the ASD situation, strategic planning and taking action to manage the situation in the province was raised:

“No, there's a better word - ignorance. It's a pervasive ignorance in the society, about the need and the problem that we're facing. So yes, you know, speaking of other things, country-wide, everything on the news at the moment, is mismanagement, poor planning, but for me it's poor planning of Eskom^*^, poor planning of resources.. but for me, it's less.. it's not just poor planning, it is a not knowing, it wasn't even aware of this coming” (P10)

^*^South African electricity utility company

Service providers raised concerns that government stakeholders were not doing enough to manage the situation despite warnings from service providers. One service provider explained:

“*so I don't know whether anybody could foresee it, but those that do…in the last six years, the same noises have been made, but I don't see a balancing. The writing was on the wall, people have been putting these things in meetings and in presentations and saying, listen, wake up, there's something coming, and I didn't see the counter to that.” (P10)*

Service providers felt that decision-makers did not acknowledge the urgency of the ASD situation. The increasing number of children being diagnosed with ASD that would require services was not taken into consideration for future planning:

“*… well maybe it's living in denial, maybe it's just simply saying, can't deal with that now, got other crisis to deal with, fighting fires within the moment, but not making long term plans…. I don't think there's enough future planning, projections made, and strategic planning toward those projections.” (P9)*

Service providers felt that there was no clear sense of who “owned” the problem and who accepted accountability and responsibility. Instead, there was a tendency to shift responsibility and not generate solutions to the problem:

“*… who's problem is it? Is it the country's problem? Is it that community's problem? Is it a social…you know, is it a worldwide problem? Is it the government's problem? Who's problem is this? And if we're now particularly talking about autism, autism is on the rise, who's problem is it? And can we shrug shoulders and say, well it's not my problem, you know, I'm doing this kind of work, that's your problem, find help for it…So we all sort of put these little blinkers on, and I know I'm making a sweeping statement, but it is easier, than to then live in the denial, and say, you know what? I'm doing the best I can, just to get my yard clean, and leave that one with the problem, to deal with their problem. Yet that person, has questions, has need for support, where… who's door, do they knock on to? I don't know, I don't have that answer. It's a philosophical one.” (P10)*

### Category 3: Building bridges

The third category described service provider acknowledgment of the efforts made by the current leadership and their suggestions for improved ASD service delivery in four sub-categories: leadership, capacity-building, co-ordination and innovation.

#### Leadership

Despite the numerous challenges alluded to in the preceding categories, service providers felt that the public sector services in the Western Cape province were taking the lead and doing much better than the rest of the country in terms of ASD service delivery:

“*Contrary to popular belief, the Western Cape is streets ahead of the rest of the country, and for many reasons. And the first reason is, the coordinated way in which things are done. There is a consolidated database (waiting list), which is maintained very efficiently, extremely efficiently, with all sorts of information about each child in that database. It's a tracking mechanism for where a child is, what happens to the child, etc., etc. It is a fair system…Fair in the sense that you obviously have to look at children first, who are school going age, those children need to be prioritized, it is first come first serve, it's not about financial status, socio-economic structure, culture doesn't play a role” (P3)*

#### Capacity-building

The expertise and experience necessary for working with children with ASD was raised. Service providers felt that increasing the competence in school staff would result in improved inclusion of children with ASD in the educational system:

“*The biggest shift they* [teachers] *need to make, is to know that in this class, you need to prepare a child for life, and not teaching math and literacy. And that's a teacher thing. Teachers are born and trained to teach math and literacy. And to make that shift, to helping you to wipe your nose, is also part of learning and getting you ready…So don't feel like you're not doing your work when you're not teaching, you're playing all day…” (P4)*

Participants proposed that the multidisciplinary educational outreach teams that were appointed by the Western Cape Education Department mainly to support the ASD schools and units across the province could support children with ASD in mainstream schools as well and advocate for better inclusion of these children in the broader education system:

“*There needs to be a service like the outreach, but it's not just for special schools, not just for screening. It should be, here we've got a service, a referral is your service, we'll come and help this teacher to include this child in her class. And that's what I was doing for nearly 2 years, and it works. But you've got to get the staff trained, you've got to get them on the same page, they're going to accept this child. And it's a whole school issue, it's whole school philosophy.” (P9)*

The provision of essential home programmes, early intervention services and family support programmes could assist in bridging the service gap while children with ASD wait for school placement:

“*I think if we could have more home programme type things, like they have here at* [an ASD school], *that would maybe be useful. So then while children are waiting on the waiting list, they at least have some intervention in the meantime as well, or some extra support…If it was an ideal world also supporting the whole family, because often I think parents struggle, the siblings struggle, so the whole family could be supported. That would be ideal” (P5)*

#### Planning and coordination

Participants advocated for long-term planning of sustainable solutions that ensured coordinated intersectoral actions to provide educational opportunities for children with ASD:

“*In the Western Cape, our biggest drawback is planning. I think we are all doers. It feels like everyone wants to jump in and do, and I'm not a planner, that's maybe why it's frustrating for me as well, I'm also a doer, I see the problem, I want to fix it. But we need to take a step back, because especially, I've looked on the waiting list yesterday, especially with the young ones coming, that are identified, is someone planning for what's coming? Is someone looking at what is on their way? Or are we just trying to figure out what to do with the ones that we have now?” (P4)*

“*Research has shown, where the best services across the world is rendered, is where you have very close inter-departmental collaboration. And that is, I think, one of the reasons why we struggle, it is almost non-existent.” (P3)*

#### Innovation

Participants advocated for innovation that would design contextually-relevant educational opportunities for every child with ASD to be among their typically-developing peers. They felt that innovative ideas could emerge from inter-professional collaboration in the design of early intervention packages drawing on international best practice examples:

“*If it's an ideal world, I'd like to have a child in a school…and the neurotypical children need to learn about how to engage with an autistic child. However, there are those children that need, and if you're asking ideal world, then I'm saying there must be way more facilities. Not only big schools with three hundred of these children together; smaller, small facilities with fifty children in, ten classes of five each, you know.” (P9)*

Service providers acknowledged that ASD is a particularly challenging condition to manage and by drawing on the experiences of other countries, the Western Cape could develop a contextually relevant model for ASD service delivery as one service provider explained:

“*I think, with intelligent people who know the landscape, to craft something that is necessarily pragmatic. There are going to be disappointments, it's an incredibly challenging condition, that the most developed countries in the world don't get right. But what are the core things that we can draw in, and build on.... let's make a concerted effort, to go look at international trends of developed countries, where they have trialed, and tried, and tested, and what is working and what does not work.” (P10)*

### Recommendations from participants for service strengthening of ASD education in the Western Cape

Table 3 shows a summary of the ten key recommendations made by participants for service strengthening of ASD education in the province. Narrative comments on these will be incorporated into the discussion.

**Table 3:** Participant recommendations for service strengthening of ASD education in the Western Cape.

**No**.	**Service provider recommendations**
1	Introduce early intervention and support to children younger than compulsory school going age on the waiting list
2	Support children and their families at home or in the community long before children require formal education
3	Rethink inclusive education so that the majority of children with ASD may not require special school placements
4	Change the stigma and perception about autism in mainstream education at primary, secondary and tertiary level
5	Develop training programmes for educators to enhance their skills to work with learners/students with ASD
6	Rethink curricula for children with ASD to be flexible, based on the needs of individual learners/students, and, where appropriate, focus on a meaningful range of life skills
7	Include users/carers and people with ASD into policymaking, curriculum development and training at all levels
8	Balance the need for more special educational provisions and improved access to mainstream settings for children with ASD
9	Avoid “knee-jerk” responses to service needs by development of long-term, integrated policies, plans and actions
10	Learn from international best practice examples to develop contextually appropriate solutions to meet the educational needs of children with ASD in South Africa

## Discussion

The purpose of this study was to examine “software” elements of ASD educational services in the Western Cape province of South Africa that might firstly, complement the “hardware” data reported to date and secondly, inform systems strengthening through service re-organization, policy review, and the development of best practices in ASD services. Earlier studies of the hardware of ASD educational services in the Western Cape province of South Africa identified a range of structural challenges – low identification rates of ASD, low identification of co-occurring diagnoses, complicated and inconsistent pathways to diagnoses and, concerningly, an observation that 89% of all children in school with a known diagnosis of ASD were in special school settings (25). In addition, the authors identified a large “waiting list” of children in need of special educational ASD placements, more than a quarter of which were of legal school-going age (26).

Using qualitative data from ten highly diverse ASD experts in the province, the findings suggested that participants perceived ASD services in the Western Cape as doing “the best we can to bridge the gap” despite the complexities of ASD population needs and prevailing contextual circumstances. However, in spite of doing “the best we can”, the majority of participants expressed significant concern about various hardware (e.g., limited human resources, infrastructure, training) and software (e.g., lack of priority of ASD and other disabilities, knee-jerk responses, ignoring early intervention) elements and made a number of recommendations to strengthen education services in the province.

Participants acknowledged the complexity of the autism spectrum that inherently poses unique service delivery challenges. Vohra and colleagues (33) concurred that individuals with ASD experience more barriers in accessing services compared to individuals with other developmental disabilities or mental health conditions. Lai and Weiss (34) argued that the variable and lifelong nature of ASD makes planning for services challenging, pointing out that individuals with ASD have normative age-dependent service needs, including timely access to identification and diagnostic services, early intervention, school services, after-school and adult services. The participants in this study felt that all levels of services for individuals with ASD were lacking in the Western Cape.

Services providers pointed out that children were on a waiting list but that there were no systems in place to provide intervention or support for these children and their families while they waited. Studies have shown that early identification, diagnosis and intervention is essential for minimizing some of the core features of ASD and thereby positively influencing the functional trajectory of the disorder throughout schooling into adulthood (35, 36). The lack of early intervention services during the critical period of development has significant consequences for language, social and cognitive development with more financial costs relating to long term care and poor prospects of employment (5). A waiting list initiative to find and intervene in young children with ASD could be a very powerful strategy for improved future outcomes.

Service providers suggested that young children with ASD should receive services either at their homes or an interim place should be provided for them to receive developmentally appropriate stimulation. There is growing evidence internationally on the effectiveness of caregiver mediated interventions (37–39). In a study from the Western Cape province of South Africa, Guler et al. (40) suggested that contextually relevant and sustainable caregiver-led interventions could bridge the service gap in low-income countries where intensive early intervention programmes are not financially accessible to the majority of the population. Furthermore de Vries (14) argued that caregiver-led interventions such as naturalistic developmental behavioral interventions (NDBIs) would be ideal for LMIC contexts where the need is far greater than the number of “expert” service providers available to deliver interventions. Therefore foregrounding policy initiatives and service efforts that target early intervention will contribute to bridging the current education service gap in the long term.

Apart from the need for early action, the ASD diagnosis also influences access to development opportunities throughout the lifespan. Critical to these opportunities is the mainstream participation and inclusion of people with disability in learning environments. Hehir et al. (41) reported on the short- and long-term benefits of inclusive education for people with disabilities: improved social and cognitive development as well as better opportunities for further education and employment. In the study by Pillay, Duncan and de Vries (25) 89% of the school-going population of children with ASD in the Western Cape attended schools for children with special educational needs and only 10% were in ordinary mainstream schools. An important perspective held by the service providers that a significant proportion of children with ASD in special schools could potentially be better placed in mainstream education supports the need for action to identify and shift children with ASD in special schools to mainstream schools. This however would require advocacy and training to facilitate successful inclusion of these children in mainstream schools.

Service providers felt that a label of ASD was associated with “fear” in educators and poor inclusion of children with ASD in mainstream schools. Nah and Tan (42) reported that caregivers seeking mainstream education services for their children with ASD in Singapore were hesitant to disclose the diagnosis of ASD due to fear of stigma and perceived negative attitudes of educators. Studies have shown that mainstream educators tend to have negative perceptions of teaching children with ASD due to lack of competence in managing social, communication and behavioral issues associated with ASD (43). According to Simpson et al. (44) a diagnosis of ASD warrants highly qualified educators with sound knowledge of evidence-based practices. They suggested that higher education institutions like universities and educator training colleges should work collaboratively with schools to improve the scope of training programmes to prepare educators for working with children with ASD (44).

A diagnosis of ASD also warrants curriculum content that is shaped by knowledge of the educational needs of this population (44, 45). Service providers in the current study expressed concerns that the skills taught to children with ASD in special schools were “mundane” and not optimal for development of their full potential. ASD special schools in the Western Cape do not follow a prescribed curriculum, only some of the schools work with individualized education and development plans (IEDPs) and children leave school at the age of eighteen without a national certificate and with limited vocational opportunities. In South Africa there are no policies specific to what children with ASD should learn, how they are taught and where they should be educated. Service providers felt that those responsible for developing special needs education policies and curricula had little or no knowledge of ASD and that the people with the necessary educational expertise and experience were not consulted. In Canada, after decades of conflict with policy makers, parent groups, with the help of researchers were able to influence policy, improve service delivery including customized programmes for their children with ASD (8). The participants in this study advocated for differentiation of the curriculum taking into account the heterogeneity of ASD. The development of a customized and national qualification aligned curriculum for learners with ASD would go a long way toward addressing the current service gaps identified in this study.

Taking into consideration the many contextual constraints that service providers described, participants acknowledged the need for an appropriate range of infrastructure and resources to provide educational opportunities for every child with ASD according to their profile of needs. At the time of this research, the Western Cape was the only province in South Africa that had a list of children with ASD waiting for a place to become available in a special school. Although the waiting list has been contentious as outlined in comments from participants, others remarked that the waiting list itself was an important resource for identifying infrastructure and service gaps. Information about the number of children waiting, their ages and other socio-cultural factors could serve to inform future planning.

Service providers felt that the lack human and infrastructural resources, compounded by the lack of competence in decision makers could negatively impact educator wellbeing. With special schools under pressure from decision-makers to admit more children with ASD in a class as a means to alleviate the waiting list, service providers expressed concerns that educators would experience “burn out”. Brunsting et al. (46) raised similar concerns about the wellbeing demands placed on ASD educators in specialized education settings. They argue that having more children with ASD in a class can lead to educator burn out despite educator's having the knowledge and skills to work with children with ASD. Mrstik et al. (47) reported that retention of educators of children with ASD in the special education sector was a major problem in the United States as well as other countries around the world resulting in a shortage of special educators. In a phenomenological study on the lived experience of educating students with ASD, educators reported that high workloads coupled with poor support from administrators was a major source of stress in educators (47).

While some service providers felt that more special schools should be provided for children with ASD others felt that greater efforts should be made to ensure successful inclusion of children with ASD in existing mainstream schools. In a study that explored the challenges and facilitators of mainstreaming children with ASD, Lindsay et al. (48) adopted the Lipsky and Gartner (49) model to inform their analysis. The model identified both human and financial resources as essential elements for inclusion (49). Similarly, Simpson et al. (44) argued for the benefits of allocating resources to address the increasing prevalence of ASD in contemporary societies, pointing out that policymakers should make financial resources available for professional development programmes and other resources that would allow mainstream classrooms to become more conducive to learning for children with ASD. The Western Cape is relatively well-resourced in comparison to other provinces (with two of the five ASD specific special schools in South Africa and several satellite ASD unit classes attached to special schools across the province), however some of the service providers felt that taking into consideration the increasing number of children being identified with ASD and the significant backlog of children already waiting for ASD educational services in the province, a range of appropriate educational opportunities for children with ASD should be explored and supported in order to bridge the service gap.

Service providers also commented on the need to learn from international good practice in order to develop contextually relevant local innovations to “build the bridges” between the current and future service landscape for children with ASD in the province. Service providers felt that greater efforts should be made for long-term planning rather than “knee-jerk” or reactionary decision making. Lessons from policymakers in Canada has shown that rushed decision-making due to political pressures resulted in poor outcomes for children with ASD and a more proactive rather than reactive approach was prescribed (8). Stronger intersectoral and inter-professional collaboration was advocated. Service providers felt that where collaboration between the different stakeholder groups was happening there was progress therefore calling for larger scale collaborative efforts. According to Cloet et al. (50) collaboration is key to meeting the diverse needs of children with ASD. Finally, service providers suggested that aligning with international ASD best practice frameworks and drawing on key concepts could support the development of contextually relevant policies and practices for the South African context.

### Limitations and future research

We acknowledge a range of limitations of this qualitative study. First, this study was conducted in one better resourced province in South Africa, and in one sub-Saharan African country. Caution should therefore be taken to generalize findings to other provinces and other LMIC. However, the high level of concern expressed about ASD education in this South African province suggests that even greater challenges may present in other South African provinces and in other LMIC. Second, although the interviews were conducted primarily by the first author, all the authors were involved in developing the interview schedule and analysing the data. Third, results reflected the perspectives of a small group of ASD service providers and findings may therefore not be easily generalisable. However, the participants represented a broad range of perspectives, and many years of collective expertise and experience of the educational system in the province. Findings are therefore likely to be a fair representation of the state of education systems in the Western Cape. Fourth, data were collected in 2018, and there may have been some “hardware” and “software” improvements since the study. However, no major changes have been observed by the authors since the data collection for this study. More research would be necessary to determine the extent to which these improvements have impacted service delivery for children with ASD and their families. Fifth, our study focused on perspectives of service providers. It will also be important to seek the views of caregivers of children with ASD, and of government stakeholders in order to ensure a multi-level evaluation of the ASD educational landscape in the province.

## Conclusion

Despite the many complex challenges of delivering educational services to children with ASD and their families in the Western Cape province of South Africa, the overarching message from participants was that everyone was doing the best they could. Service providers felt that services could improve if collaborative efforts were made by different stakeholder groups to understand and strengthen education systems. Educator training to facilitate inclusive education for children with ASD in the greater education system, parent-mediated early intervention, and intersectoral and inter-professional collaboration were identified as areas that could bridge the service gap. Drawing on international frameworks to develop contextually appropriate ASD policies and best practice for South Africa were recommended.

## Data Availability Statement

Enquiries about the primary data can be directed to the corresponding author.

## Ethics Statement

Ethical approval was granted by the University of Cape Town Human Research Ethics Committee (HREC reference: 072/2016) as well as the Western Cape Department of Education (reference: 20150727-1712).

## Author contributions

SP, MD, and PJDV participated in the conception and design of the study. SP collected the data. MD and SP were involved in the first level of data analysis, consensus on the final categories, and themes were reached through discussions with all the authors. All authors approved the final version of the manuscript.

## Conflict of interest

The authors declare that the research was conducted in the absence of any commercial or financial relationships that could be construed as a potential conflict of interest.

## Publisher's note

All claims expressed in this article are solely those of the authors and do not necessarily represent those of their affiliated organizations, or those of the publisher, the editors and the reviewers. Any product that may be evaluated in this article, or claim that may be made by its manufacturer, is not guaranteed or endorsed by the publisher.
